# Multimodal Rehabilitation Management of a Misunderstood Parsonage–Turner Syndrome: A Case Report during the COVID-19 Pandemic

**DOI:** 10.3390/jfmk9010037

**Published:** 2024-02-23

**Authors:** Fabio Santacaterina, Marco Bravi, Mirella Maselli, Federica Bressi, Silvia Sterzi, Sandra Miccinilli

**Affiliations:** 1 Department of Physical and Rehabilitation Medicine, Fondazione Policlinico Universitario Campus Bio-Medico, Via Alvaro del Portillo, 200, 00128 Rome, Italy; m.bravi@policlinicocampus.it (M.B.); m.maselli@policlinicocampus.it (M.M.); f.bressi@policlinicocampus.it (F.B.); s.sterzi@policlinicocampus.it (S.S.); s.miccinilli@policlinicocampus.it (S.M.); 2 Research Unit of Advanced Robotics and Human-Centred Technologies, Department of Engineering, Università Campus Bio-Medico di Roma, Via Alvaro del Portillo, 21, 00128 Roma, Italy; 3 Research Unit of Physical and Rehabilitation Medicine, Department of Medicine and Surgery, Università Campus Bio-Medico di Roma, Via Alvaro del Portillo, 21, 00128 Roma, Italy

**Keywords:** Parsonage–Turner syndrome, brachial plexus neuritis, rehabilitation, shoulder, physiotherapy, referral

## Abstract

During the second wave of the COVID-19 pandemic, a young adult presented symptoms that were reported at first evaluation to be a frozen shoulder (adhesive capsulitis). The patient’s history, clinical manifestations related to the onset of pain, unilateral weakness, and physical examination led to a physiotherapy referral. Subsequent instrumental investigations showed an idiopathic brachial neuritis known as Parsonage–Turner Syndrome (PTS). Contrary to recent descriptions in the literature, the patient did not experience PTS either after COVID-19 vaccination or after COVID-19 virus infection. The proposed multimodal treatment, considering the patient’s characteristics, led to a recovery of muscle strength and function of the upper limb, observed even three years after the acute event. The frequency of rehabilitation treatment, the choice of exercises, the dosage, and the methods of execution require further studies in order to define an evidence-based treatment.

## 1. Introduction

PTS is defined as a peripheral nervous system disorder characterized primarily by two key elements: severe pain and significant muscular atrophy in the shoulder complex [1]. The pain in this unilateral acute plexopathy tends to diminish within a few days or at most two weeks, giving way to marked muscular atrophy, motor paralysis, and sensory deficits [2]. The pathophysiological mechanisms underlying PTS are not entirely clear but appear to involve genetic, immunological, and biomechanical factors [3]. Some authors hypothesize an association between PTS and viral infections, surgery, and vaccinations [3]. A recent systematic review analyzed 26 reported cases of PTS following the administration of the SARS-CoV-2 vaccine [2]. Another systematic review suggested an association not only with COVID-19 infection but also after vaccination, with an earlier onset of PTS symptoms post-vaccination compared to the infection itself [4]. It typically affects adult males more frequently with a sudden onset [5], and sometimes atypical manifestations can occur, such as lower limb and diaphragmatic deficits [6]. The shoulder muscle deficit leads to compensatory movements and scapular dyskinesia, which, while initially useful, can result in a persistent altered pattern and movement limitation at the long term [7]. This aspect is crucial since 60% of patients have residual weakness 2–3 years after the onset of the disease; over 50% are limited by pain; 63% experience severe fatigue; and 82% face difficulties in daily activities [8]. Several strategies have recently been proposed for the rehabilitation of PTS patients, such as a structured multidisciplinary approach that has proven effective [9,10]. This case report shows, for the first time, through an objective strength evaluation system, how recovery can occur even after three years and the role that a progressive multimodal rehabilitation treatment can have in achieving outcomes.

## 2. Materials and Methods

### 2.1. Case Report

This case report, written in line with CARE guidelines [11], describes the diagnostic and rehabilitation process of a 36-year-old Caucasian male, born and raised in Italy, employed in the financial sector, and regularly engaged in physical activities (three gym sessions weekly). He had a history of right wrist fracture surgery (2008), left knee anterior cruciate ligament reconstruction surgery (August 2020), and removal of a lipoma in the left cervical region in 2019. The patient had no comorbidities and was in excellent general health before the acute event.

### 2.2. Clinical Findings

**Figure 1:**
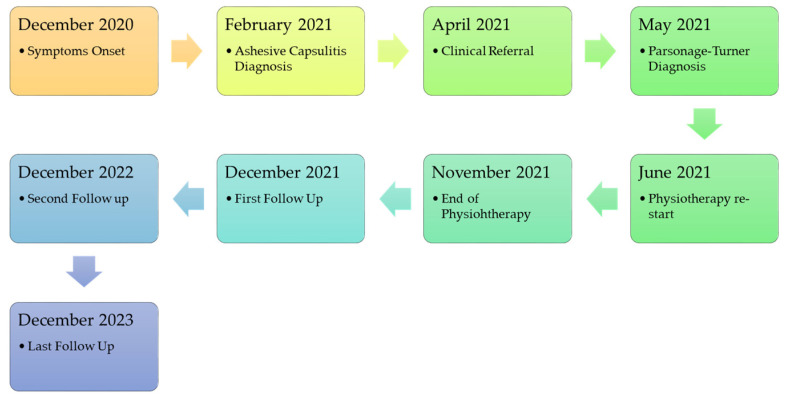
Case report timeline.

Since December 2020, the patient reported minimal shoulder pain and mild difficulty in right shoulder movement (dominant upper limb). In January 2021, he woke up with severe right shoulder pain (8/10 Numeric Pain Rating Scale—NPRS), unable to raise his arm beyond 90° in active flexion and abduction, as reported from the patient. After an inconclusive shoulder magnetic resonance imaging (MRI), prescribed by a general practitioner on 24 January 2021, he consulted an orthopedic physician in February 2021, who diagnosed “initial adhesive capsulitis of the right shoulder in the context of scapula–humeral girdle mispositioning due to incorrect posture”. Cortisone and hyaluronic acid injections were administered, along with ten individual motor rehabilitation sessions and additional therapies (Tecar^®^ therapy, ultrasound therapy, and low-level laser therapy). Despite the prescribed treatment, the patient showed no improvement after the initial ten sessions and attracted the authors’ attention in April 2021 (Figure 1: case report timeline).

### 2.3. Diagnostic Assessment

**Figure 2:**
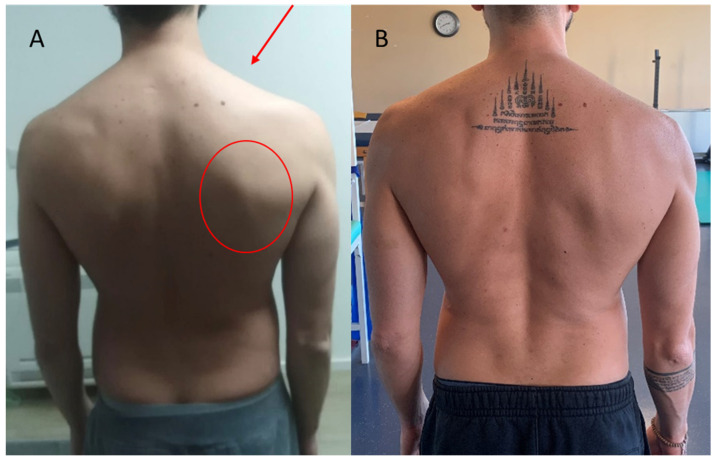
(**A**) Posterior view with scapular inferior angle prominence (red circle). The red arrow shows the upper trapezius hypotrophy, February 2021. (**B**) Posterior view, December 2023.

A subsequent new physiotherapy assessment on April 2021, through an interview with the patient, observation, and evaluation, revealed scapular alteration [12] (Figure 2A), persistent muscles weakness with marked hypotrophy of the right upper trapezius and sternocleidomastoid, altered shoulder dynamics, and full passive shoulder range of motion (ROM), not found in a typical frozen shoulder 2 months after painful onset, leading to a referral by the physiotherapist to a neurologist in May 2021. The neurologist suspected accessory nerve injury and ordered electromyography (EMG) and cervical plexus MRI. The EMG indicated reduced recruitment with polyphasic potentials and mild fibrillation involving the accessory nerve, long thoracic nerve, and suprascapular nerve. Cervical plexus MRI showed minimal thickening and heterogeneity of the lateral, medial, and posterior cords, suggesting mild neuropathy. The neurologist recommended discontinuing physiotherapy for a month, corticosteroid therapy for one month, and then reassessment. EMG follow-up (June 2021) showed improved nerve conductivity, allowing for the resumption of physiotherapy. The new physiotherapy evaluation, on June 2021, showed an improvement in the performance of the affected muscles, even if still deficient, through muscle evaluation with the Medical Research Council (MRC) (trapezius upper fibers 3/5, serratus anterior 3/5, sternocleidomastoid 4/5) and a reduction in painful symptoms that persisted in the last 20° of active shoulder flexion and last 10° of active shoulder abduction (3/10 NPRS). The patient still reported persistent pain even at rest, especially at night (2/10 NPRS).

### 2.4. Therapeutic Intervention

The resumption of rehabilitation activity in June 2021 took into account the long diagnostic process of the patient who experienced periods of frustration regarding the persistence of muscles weakness and functional deficits. We therefore planned to modulate pain through manual therapy in the short term (Table 1). Furthermore, a triple application of elastic taping, previously used in the literature for reducing shoulder pain and improving function [13], was applied in the short term on a three-weekly basis. At the same time, muscle strengthening activity was resumed through therapeutic exercise to achieve the medium-term objective of recovering muscle strength (Table 1). To this end, we proceeded with the execution of open and closed kinetic chain exercises of the target muscles (Table 1). The exercises were chosen considering the patient’s preferences. The long-term goal was to bring the patient back to resuming his sporting activity (body building) safely and without difficulty (Table 1). An extended description of the treatment protocol is available in Appendix A. The patient was followed until November 2021 with an intensive physiotherapy program. After this date, he continued with home exercises and gym activity on his own. Follow-ups were then carried out up to 3 years after the acute event.

## 3. Results

The patient underwent several follow-ups through physiatry and neurological check-ups to monitor clinical conditions during the rehabilitation period. A new EMG examination (September 2021) reported improvements in the conduction of the affected nervous structures with the persistence of slight signal alterations. A physiatric evaluation (September 2021) also reported clinical improvement with the resolution of pain on movement and at rest (0/10 NPRS), full active shoulder ROM, and the persistence of shoulder muscle strength deficit (trapezius upper fibers 4/5, serratus anterior 4/5, sternocleidomastoid 4/5 MRC), and the need for physiotherapy sessions, performed until November 2021. After the rehabilitation process (12, 24, and 36 months from the acute event), the patient underwent strength tests with a handheld dynamometer (DynaMo, VALD Performance, Australia), with the patient standing upright (Table 2), and administration of the DASH, a patient-reported outcome measure (PROM) (Table 3). The data show how recovery occurred slowly over the 3 year follow-up. Currently the patient has returned to carrying out all the activities of daily life and has reintegrated without problems into his social and working context. He returned to sporting activity in the gym. Furthermore, scapular alteration was no longer appreciable in posterior vision at the December 2023 follow-up (Figure 2B).

## 4. Discussion

PTS is a more prevalent condition than has been previously estimated [14]. A prospective study suggests an incidence of 1 in 1000 [15], confirming that, probably due to its deceptive manifestation, it is underestimated.

The literature indicates that PTS should be considered in diagnostic hypotheses for patients with severe pain and weakness following COVID-19 vaccination [16]. In our specific case, despite occurring during the peak of the second pandemic wave, the patient had not received any vaccine doses and had not contracted the virus. It is our opinion that COVID indirectly influenced a characteristic aspect of this patient: before the physiotherapy referral, which occurred in April 2021, the patient had never been observed without a t-shirt, both in medical evaluations and during physiotherapy treatments. This could be attributed to distancing-related hesitations and fears of possible contagion experienced during the pandemic. An initial lesson from this case report is undoubtedly the importance of observation, inspection, and palpation during the patient’s physical examination, which should always be preceded by a thorough initial conversation. The speed with which some specialist consultations occur, without paying attention to clinical manifestations, such as typical unilateral muscle hypotrophy, scapular alteration, and full passive ROM, can lead to inappropriate therapeutic choices and delay the patient’s recovery. It is essential to consider how PTS can mimic a common shoulder pathology, thus confusing the diagnostic algorithm. It should also be highlighted that the onset with severe pain is common to both PTS [2,5] and frozen shoulder [17,18]; this could therefore have misled the first diagnosis. In fact, the patient, at the first evaluation, reported a high pain level (8/10 NPRS). Furthermore, the scientific literature has already shown how the two pathologies can sometimes be confused [5]. It is also crucial to emphasize the timing in PTS diagnoses; in many cases, denervation is no longer easily identifiable with MRI or EMG after 2–4 weeks [16]. The diagnostic timing in this case report is characteristic, showing that, despite various consultations, a diagnostic hypothesis was only considered after several months. Additionally, clinical presentation, physical examination, and diagnostic tests are useful for differentiating PTS from cervical radiculopathy, thereby reducing healthcare costs and avoiding inappropriate procedures and treatments [19].

The characteristic element of this case report was the proposed rehabilitation treatment; the multimodal approach, related to timing and short-, medium-, and long-term goals, appears to have contributed to the recovery of strength and function of the upper limb. Currently, in the literature, the rehabilitation process lacks certainty, but some elements seem effective and are widely used, such as neurodynamic techniques [20]. Recent studies have shown that a multidisciplinary approach appears valid in managing these patients, emphasizing the importance of sensorimotor recovery, including exercises for regaining upper limb proprioceptive abilities and motor imagery [9,10]. A factor that had a positive influence on the patient’s prognosis in this case report is his young age, motivation, active lifestyle, and adherence to the therapeutic plan (the patient attended all the scheduled sessions). Patient-centered action, the consideration of preferences in exercise selection, and the constant monitoring of conditions are elements that may have contributed to the therapeutic alliance established. An element that may had a role in the recovery of strength was the introduction of modifications to the TUT depending on the specific aims; in fact, it was initially preferred to use a high TUT with low loads, so as to not exacerbate the painful symptoms. Secondly, afterwards, we moved on to increasing the loads by reducing the TUT itself, and, finally, exercises related to the return to sporting activity were carried out. Basing the exercise only on the load and the number of repetitions does not seem to be sufficient for strength recovery, and changes to the TUT may play a role [21], but further studies are necessary, as a recent systematic review has shown that it is still a poorly studied topic, especially regarding the upper limb [22]. The results obtained in the various follow-ups show how the recovery occurred over a very long period and in a progressive manner, both in terms of upper limb strength and functional recovery. Future research should focus on rehabilitation treatment modalities, timing, and exercise specificity to propose evidence-based treatment.

The patient was asked the following questions:

How do you feel about your shoulder today? How do you assess your condition? Do you feel you have returned to the same levels as before? “Currently, I feel better. I don’t think I’ve fully recovered. In the gym, I can do all the exercises, but sometimes I feel like my shoulder ‘slips away’, especially when I’m more tired. Also, when I see myself in the mirror, I notice that my right shoulder is more forward than the other. I feel the strength has returned, but when I’m more tired, I feel I need more control over the body.”

What idea have you formed about what you have experienced in these two years regarding the shoulder disorder? “I think I have to learn to live with these small disturbances that remain. I’m continuing to go to the gym because I believe the condition can still improve”.

## 5. Conclusions

PTS can have an insidious onset and hide behind more common pathologies. Observation, inspection, and physical examination are fundamental elements to evaluate the presence of this condition. Recovery, often not complete, may occur even after three years. The proposed multimodal treatment represents a valid alternative to managing this pathological condition. Further studies are needed to define a fully evidence-based action.

## Figures and Tables

**Table 1:** Rehabilitation aims and therapeutic strategies carried out during the rehabilitation process.

Timing	Short Term (within 1 Month)	Mid Term (within 3 Month)	Long Term (within 6 Month)
Aims	Pain modulation and recovery of muscle strength	Complete recovery of muscle strength and movement patterns	Return to sport
Weekly sessions	5 times a week	3 times a week	2 times a week
Treatments	- Myofascial release of the target muscles - Neurodynamic techniques for mechanosensitivity - Shoulder elastic taping - Pompage techniques of the cervical spine, trapezius, sternocleidomastoid, and levator scapulae. - Mobilization with Movement (MWM) for the recovery of the last degrees of flexion and abduction in the absence of pain - Manipulations of the dorsal spine - Therapeutic exercise for shoulder ROM recovery	- Open kinetic chain exercises with progression of intensity, frequency, time under tension, and load parameters - Closed kinetic chain exercises with progression of intensity, frequency, time under tension (TUT), and load parameters - Joint position sense exercise for the upper limb - Exercises using TRX to improve target muscle activation	- Progressions for recovering the movements requested by the patient: push up, pull up, plank, side plank, deadlift, squat

**Table 2:** Shoulder muscle dynamometer test follow-up results at 12, 24, and 36 months. All force data are normalized to weight (N/Kg).

Dynamometer Shoulder Tests	Affected Side [Right]			Healthy Side [Left]		
	12 Months	24 Months	36 Months	12 Months	24 Months	36 Months
Flexion [N/Kg]	0.88	1.08	1.28	0.95	0.92	1.13
Extension [N/Kg]	2.10	2.18	2.10	1.89	1.93	1.80
Abduction [N/Kg]	0.94	1.09	1.34	1.01	1.10	1.08
Adduction [N/Kg]	1.55	1.86	1.78	1.49	1.62	2.08
Internal rotation [N/Kg]	0.85	0.99	1.03	1.25	1.15	1.19
External rotation [N/Kg]	0.78	0.90	1.03	1.10	1.15	0.99

**Table 3:** DASH follow-up results at 12, 24, and 36 months for the affected side.

DASH Section	12 Months	24 Months	36 Months
DASH principal (%)	64.2%	45%	21.7%
DASH optional (%)	87.5%	75%	40.6%

**Figure A1:**
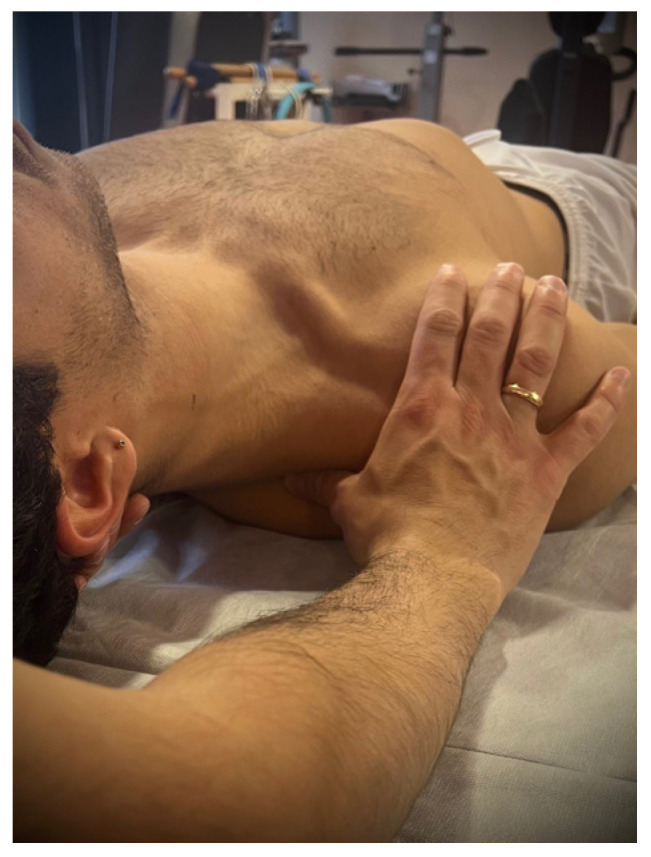
Myofascial release of upper trapezius muscle.

**Figure A2:**
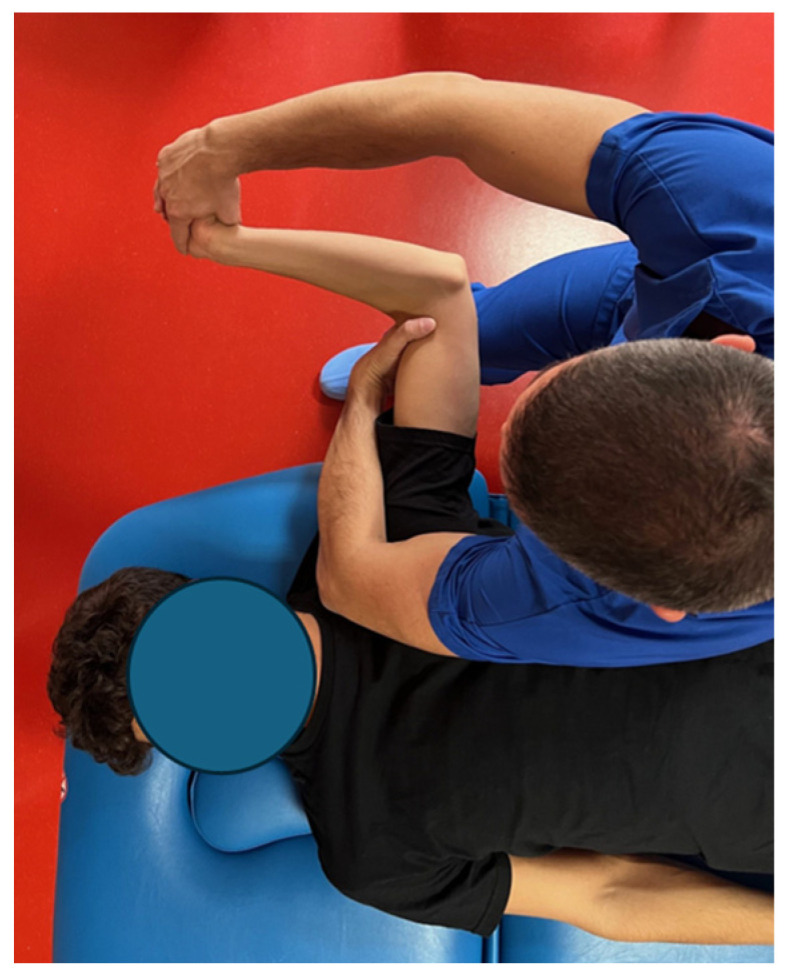
ULNT1 Neurodynamic technique for mechanosensitivity.

**Figure A3:**
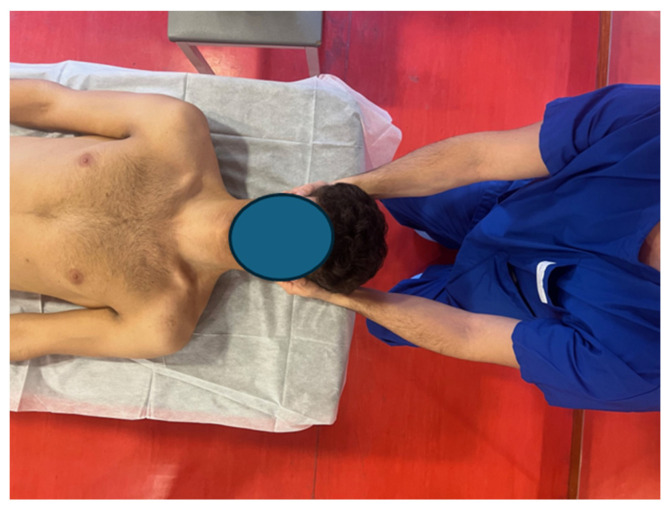
Pompage technique for cervical spine.

**Figure A4:**
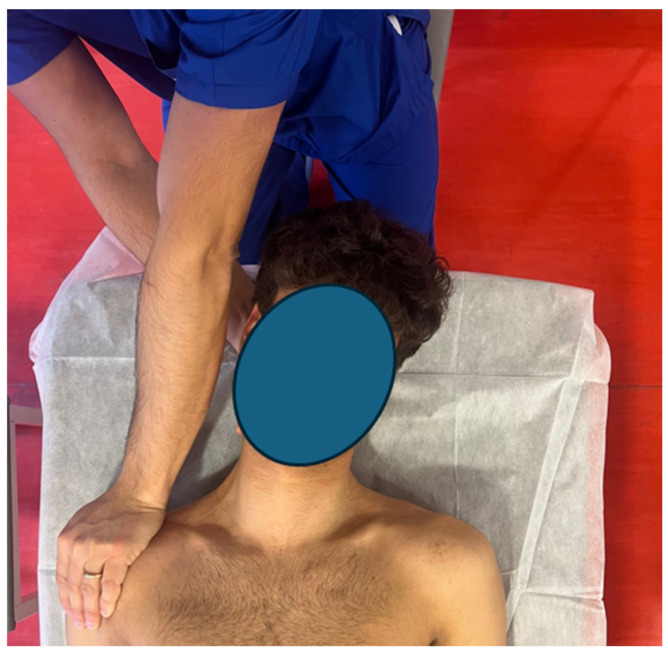
Pompage technique for upper trapezius muscle.

**Figure A5:**
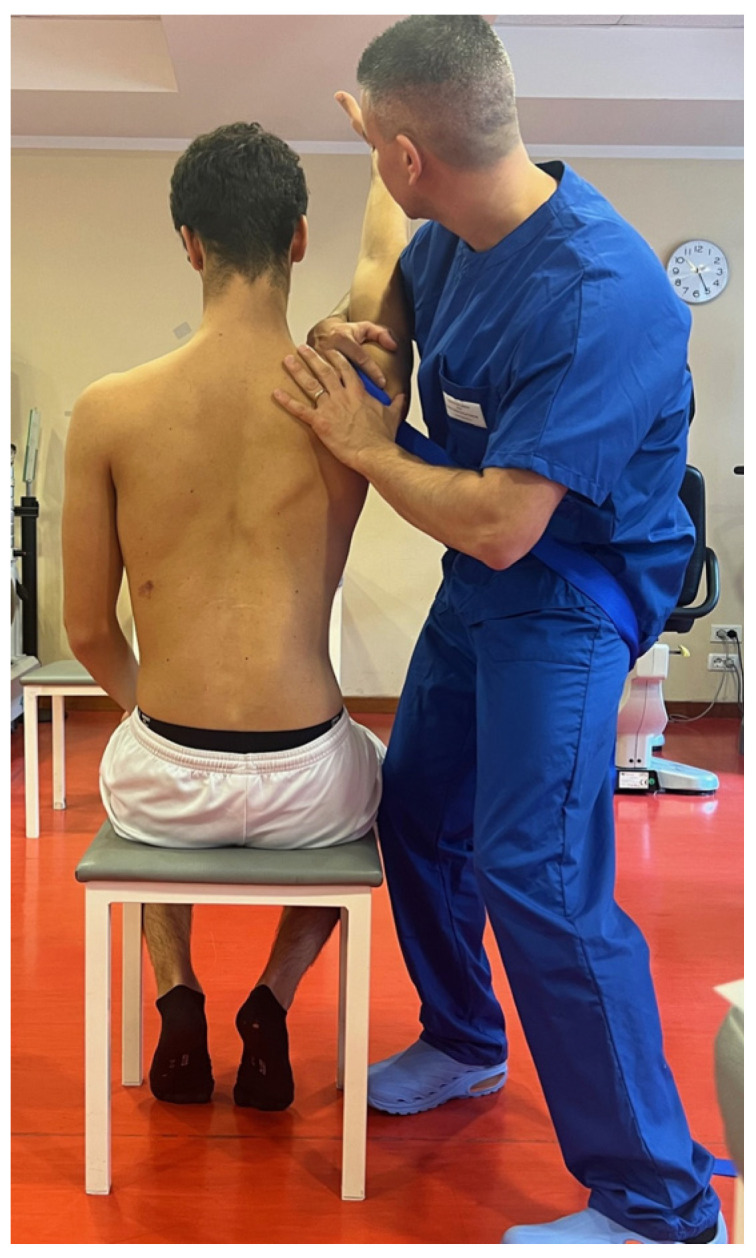
MWM for shoulder flexion recovery with a Mulligan belt.

**Figure A6:**
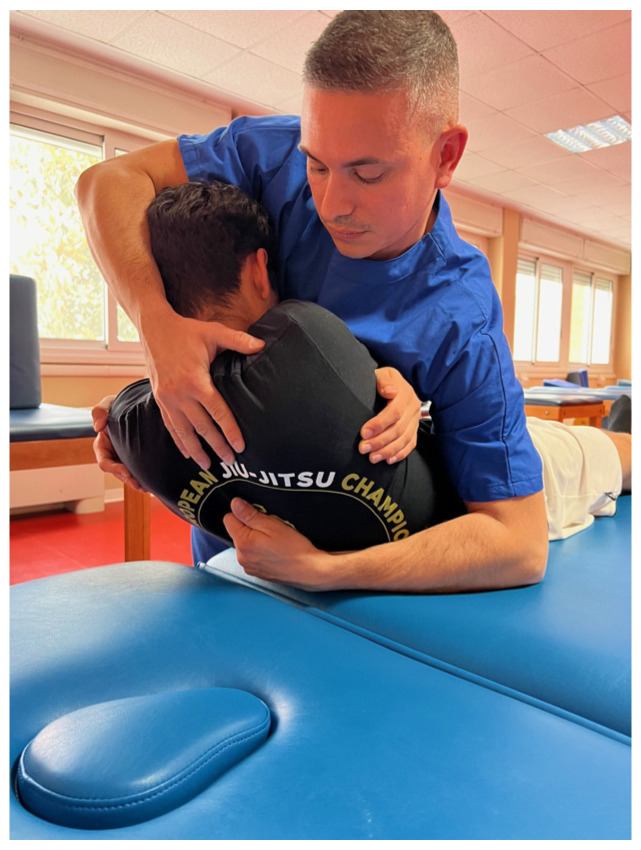
HVLA technique for the dorsal spine.

**Figure A7:**
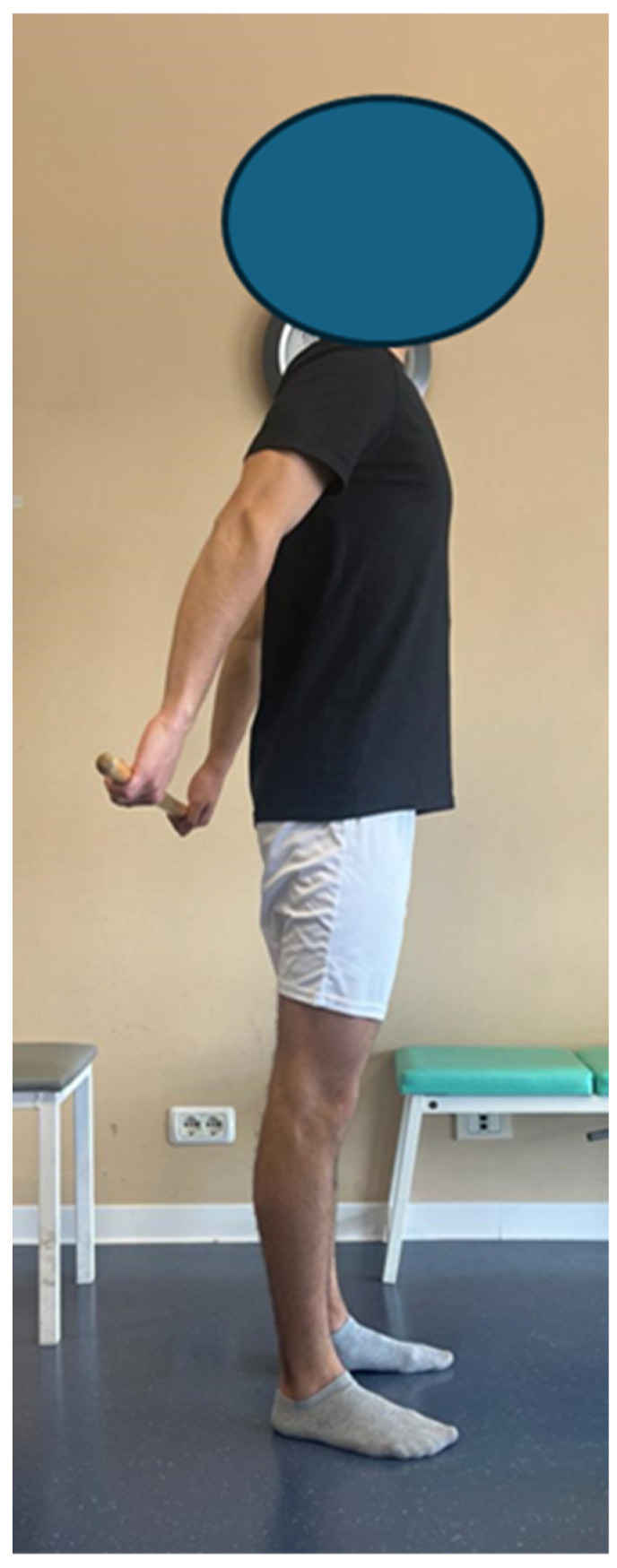
Shoulder extension exercise with a stick.

**Figure A8:**
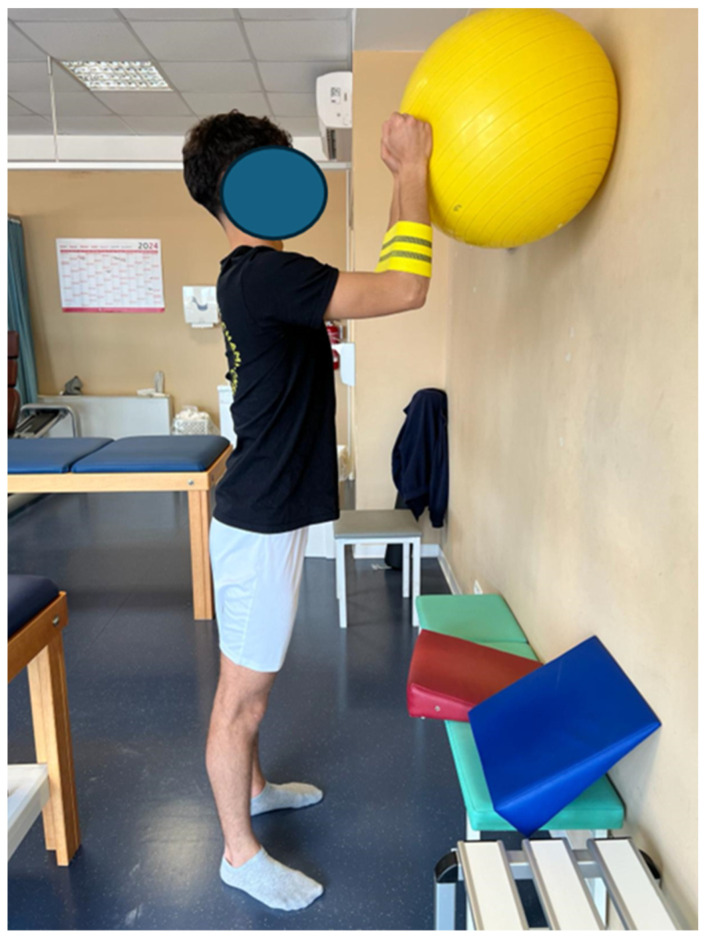
Shoulder flexion sliding wall exercise with an elastic band on the forearms.

**Figure A9:**
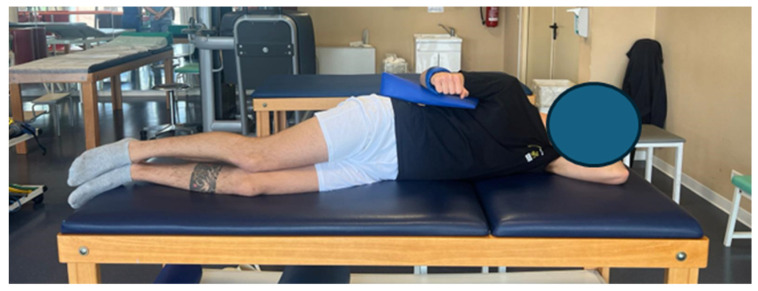
Loaded shoulder external rotation exercise (open kinetic chain).

**Figure A10:**
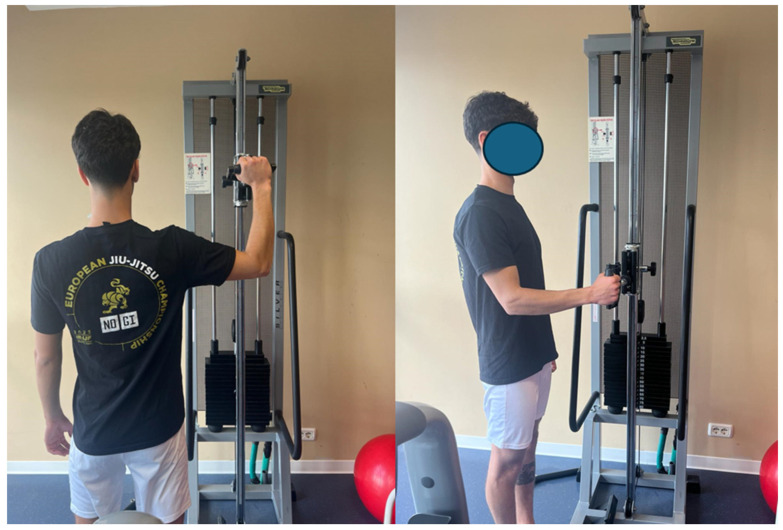
Loaded shoulder external rotation exercise (closed kinetic chain).

## Data Availability

All data relating to quantitative evaluations are reported in the text. For privacy reasons, it is not possible to attach medical reports.

## Appendix A

This section was created to show the physiotherapy sessions. The rehabilitation protocol was divided based on short-, medium-, and long-term aims. The short-term goal was to modulate the pain and recover the shoulder’s active ROM. The sessions were carried out on a daily basis, from Monday to Friday, for a total of 5 sessions per week. To achieve the short-term aims, it was necessary to carry out 20 sessions for a treatment period of approximately one month. Treatments were therefore proposed for the following:

- Myofascial release of the target muscles: a low-load and long-duration stretch to the barriers in the myofascial tissue is applied for modulate pain. The upper trapezius, the sternocleidomastoid, and the rhomboid muscles were mainly treated. The aim of this technique is to modulate pain. We used myofascial release for the first 5 sessions.

- Neurodynamic techniques for mechanosensitivity: The aim of this technique was to initially reduce neuro tension (slider in–out) reproducing the upper limb neural tension 1 (ULNT1) on the healthy limb, and then move on to increase neuro tension (slider out–in), reproducing the ULTN1 on the affected limb (Figure A2). The aim of this technique is to reduce neuropathic symptoms and improve neuro tension. We used neurodynamic techniques for the first 10 physiotherapy sessions.

- Shoulder elastic taping: We used this technique in the short term, as described in the literature [13].
- Pompage techniques of the cervical spine, trapezius, sternocleidomastoid, and levator scapulae: Pompage is a manual technique typically used in physiotherapy. It acts on both muscular components of the joint as well as interapophyseal areas. The technique consists of gradual, assisted tensions, which compress small intervertebral joints and stretch spinal ligaments and muscles (Figure A3 and Figure A4). The aim of this technique is to modulate pain. We used these techniques in the first 20 physiotherapy sessions.

- Mobilization with movement (MWM): According to the Mulligan Concept, the aim of this technique is to improve range of motion thanks to the application of a sustained gliding force with a concurrent active movement performed by the patient. We used these techniques in all 20 initial sessions (Figure A5).

- Manipulations of the dorsal spine: We used a high-velocity low-amplitude technique (HVLAT), known in the literature as “dog”, for the middle thoracic vertebrae for modulating pain. This technique is described as “a skilled, passive manual therapeutic maneuver during which a synovial joint is beyond the normal physiological range of movement (in the direction of the restriction) without exceeding the boundaries of anatomical integrity [23]”. We used this technique two times, at the beginning of the treatment and 14 days later.

- Therapeutic exercise for ROM recovery and motor pattern: In combination with pain modulation techniques, therapeutic exercise was used, without overloads, to recover ROM and motor patterns. To this end, for example, exercises with a stick were used to recover flexion and abduction, as well as sliding exercises on the board and then against the wall. The exercises were used for the first 20 sessions, increasing the number of repetitions based on the pain perceived during the exercise (we accepted exercises that did not exceed an NPRS of 3/10) and that had a moderate intensity (perceived exertion ratings between 12 and 14 on the Borg scale).

In September 2021, as reported in the main text, the patient reported an improvement in painful symptoms and the recovery of complete active ROM but the persistence of muscle weakness. We then proceeded with the medium-term goal, that is, the recovery of muscle strength. To this end, three weekly sessions were carried out for 2 months for a total of 18 sessions. The treatment consisted of the following:

- Open and closed kinetic chain exercises with a progression of intensity, frequency, time under tension, and load parameters: Open and closed kinetic chain exercises were used, initially considering the use of low loads (30% of 1RPM) and a high TUT (4 s for the concentric phase and 4 s for the eccentric phase), considering the same parameters used for short-term therapeutic exercise (NPRS values not exceeding 3/10 and Borg scale between 12 and 14). After approximately 10 sessions, we proceeded with the implementation of loads (up to 60% of 1RPM) and a lower TUT (2 s for the concentric phase and 2 s for the eccentric phase), using an NPRS not exceeding 3 as a parameter/10 and a Borg scale not exceeding 18–20.

- Joint position sense exercise for the upper limb: Exercises were used to improve the sense of position using a laser pointer placed first on the shoulder, then on the elbow, and finally on the hand, requiring the patient to follow paths set up on the wall.
- Exercises using TRX to improve target muscle activation: During these sessions, the patient expressed his desire to repeat some exercises that were part of his routine prior to the acute event. The TRXs were then used to reproduce some exercises that the patient previously performed in the gym to activate the posterior muscles of the back.

Once a maximum score in the MRC was achieved, we proceeded with the recovering of sport-specific movements. A total of two sessions were carried out per week for 3 weeks for a total of six sessions. The following were performed:

- Progressions for recovering the movements requested by the patient: push up, pull up, plank, side plank, deadlift, squat. The patient requested to perform the exercises he performed in his routine for his sport (bodybuilding) under the supervision of the physiotherapist. The patient was then supervised and instructed as regarded the exercises indicated.

## References

[1] Cornea A., Lata I., Simu M., Rosca E.C. (2023). Parsonage-Turner Syndrome Following SARS-CoV-2 Infection: A Systematic Review. Biomedicines.

[2] Parsonage M.J., Aldren Turner J.W. (1948). Neuralgic Amyotrophy; the Shoulder-Girdle Syndrome. Lancet.

[3] Greis A.C., Petrin Z., Gupta S., Magdalia P.B. (2014). Neuralgic Amyotrophy. Brain Nerve.

[4] Ameer M.Z., Haiy A.U., Bajwa M.H., Abeer H., Mustafa B., Ameer F., Amjad Z., Rehman A.U. (2023). Association of Parsonage-Turner Syndrome with COVID-19 Infection and Vaccination: A Systematic Review. J. Int. Med. Res..

[5] Feinberg J.H., Radecki J. (2010). Parsonage-Turner Syndrome. HSS J..

[6] Van Alfen N., Van Engelen B.G.M. (2006). The Clinical Spectrum of Neuralgic Amyotrophy in 246 Cases. Brain.

[7] Van Eijk J.J.J., Groothuis J.T., Van Alfen N. (2016). Neuralgic Amyotrophy: An Update on Diagnosis, Pathophysiology, and Treatment. Muscle Nerve.

[8] Cup E.H., Ijspeert J., Janssen R.J., Bussemaker-Beumer C., Jacobs J., Pieterse A.J., Van Der Linde H., Van Alfen N. (2013). Residual Complaints after Neuralgic Amyotrophy. Arch. Phys. Med. Rehabil..

[9] Janssen R.M.J., Lustenhouwer R., Cup E.H.C., Van Alfen N., Ijspeert J., Helmich R.C., Cameron I.G.M., Geurts A.C.H., Van Engelen B.G.M., Graff M.J.L. (2023). Effectiveness of an Outpatient Rehabilitation Programme in Patients with Neuralgic Amyotrophy and Scapular Dyskinesia: A Randomised Controlled Trial. J. Neurol. Neurosurg. Psychiatry.

[10] Lustenhouwer R., Cameron I.G.M., van Alfen N., Toni I., Geurts A.C.H., van Engelen B.G.M., Groothuis J.T., Helmich R.C. (2023). Cerebral Adaptation Associated with Peripheral Nerve Recovery in Neuralgic Amyotrophy: A Randomized Controlled Trial. Neurorehabil. Neural. Repair..

[11] Riley D.S., Barber M.S., Kienle G.S., Aronson J.K., von Schoen-Angerer T., Tugwell P., Kiene H., Helfand M., Altman D.G., Sox H. (2017). CARE Guidelines for Case Reports: Explanation and Elaboration Document. J. Clin. Epidemiol..

[12] Kibler W.B., Uhl T.L., Maddux J.W.Q., Brooks P.V., Zeller B., McMullen J. (2002). Qualitative Clinical Evaluation of Scapular Dysfunction: A Reliability Study. J. Shoulder Elb. Surg..

[13] Miccinilli S., Bravi M., Morrone M., Santacaterina F., Stellato L., Bressi F., Sterzi S. (2018). A Triple Application of Kinesio Taping Supports Rehabilitation Program for Rotator Cuff Tendinopathy: A Randomized Controlled Trial. Ortop. Traumatol. Rehabil..

[14] IJspeert J., Janssen R.M.J., van Alfen N. (2021). Neuralgic Amyotrophy. Curr. Opin. Neurol..

[15] Van Alfen N., Van Eijk J.J.J., Ennik T., Flynn S.O., Nobacht I.E.G., Groothuis J.T., Pillen S., Van De Laar F.A. (2015). Incidence of Neuralgic Amyotrophy (Parsonage Turner Syndrome) in a Primary Care Setting--a Prospective Cohort Study. PLoS ONE.

[16] Meixedo S., Correia M., Machado Lima A., Carneiro I. (2023). Parsonage-Turner Syndrome Post-COVID-19 Oxford/AstraZeneca Vaccine Inoculation: A Case Report and Brief Literature Review. Cureus.

[17] Lewis J. (2015). Frozen Shoulder Contracture Syndrome—Aetiology, Diagnosis and Management. Man. Ther..

[18] Chan H.B.Y., Pua P.Y., How C.H. (2017). Physical Therapy in the Management of Frozen Shoulder. Singap. Med. J..

[19] Silverman B., Shah T., Bajaj G., Hodde M., Popescu A. (2022). The Importance of Differentiating Parsonage-Turner Syndrome From Cervical Radiculopathy: A Case Report. Cureus.

[20] Wolny T., Glibov K., Granek A., Linek P. (2023). Ultrasound Diagnostic and Physiotherapy Approach for a Patient with Parsonage-Turner Syndrome-A Case Report. Sensors.

[21] Coratella G. (2022). Appropriate Reporting of Exercise Variables in Resistance Training Protocols: Much More than Load and Number of Repetitions. Sports Med. Open.

[22] Mcleod J.C., Currier B.S., Lowisz C.V., Phillips S.M. (2024). The Influence of Resistance Exercise Training Prescription Variables on Skeletal Muscle Mass, Strength, and Physical Function in Healthy Adults: An Umbrella Review. J. Sport Health Sci..

[23] Galindez-Ibarbengoetxea X., Setuain I., Andersen L.L., Ramírez-Velez R., González-Izal M., Jauregi A., Izquierdo M. (2017). Effects of Cervical High-Velocity Low-Amplitude Techniques on Range of Motion, Strength Performance, and Cardiovascular Outcomes: A Review. J. Altern. Complement. Med..

